# The complete chloroplast genome sequence of traditional Chinese medicine *Uncaria macrophylla* (Rubiaceae)

**DOI:** 10.1080/23802359.2022.2048215

**Published:** 2022-04-24

**Authors:** Miao Zhang, Min-Min Chen, Xue-Min Zhang, Shao-Ning Chen, Zong-Suo Liang

**Affiliations:** a College of Life Sciences and Medicine, Zhejiang Province Key Laboratory of Plant Secondary Metabolism and Regulation, Zhejiang Sci-Tech University, Hangzhou, China; b Tianjin Tasly Modern Chinese Medicine Resources Co., Ltd., Tianjin, China

**Keywords:** *Uncaria macrophylla*, chloroplast genome, phylogeny

## Abstract

*Uncaria macrophylla* (Rubiaceae) is a medicinal vine plant of the Rubiaceae family that was distributed in East Asia and Southeast Asia. The first complete chloroplast genome of *Uncaria macrophylla* was sequenced and assembled in this study. The genome is 155,138 bp in length and contained 129 encoded genes in total, including 79 protein-coding genes, eight ribosomal RNA genes, and 37 transfer RNA genes. The phylogenomic analysis showed that *U. macrophylla* was closely related to *Uncaria rhynchophylla* according to the current sampling extent.

*Uncaria macrophylla Wall.* 1824 (Rubiaceae) is a medicinal herb with double or single hooks in leaf axils. It is mainly distributed in tropical areas, concentrated in Malaysia to Solomon Islands in Southeast Asia, other parts of Asia and Africa, South America also have distribution (Ndagijimana et al. 2013). As antihypertensive, antioxidation, antipyretic, and anticonvulsant, it is used in the treatment of headache, vertigo, and epilepsy, and also has a certain effect on Alzheimer's disease (Fujiwara et al. 2006). Previous studies have shown that alkaloids in Uncaria, such as rhynchophylline and isorhychophylline, can be developed as chemical drugs for the treatment of human hypertension (Wang et al. 2019). In this study, the first complete chloroplast (cp) genome of *U. macrophylla* was reported. To provide genomic resources for investigating the evolution of *U. macrophylla*, the complete cp genome of this species was analyzed from high-throughput Illumina sequencing reads. It will provide a better understanding on the evolution and genetics of Rubiaceae.

The leaves of *U. macrophylla* were collected from Nanning, Guangxi, China (GPS: E108°30′3.22″, N22°47′53.21″). The specimen was deposited at Zhejiang Province Key Laboratory of Plant Secondary Metabolism and Regulation (http://sky.zstu.edu.cn/), in Zhejiang Sci-tech University, under the voucher number ZSTU09554 (Miao Zhang: Zhwater1063@163.com). Permission was granted by Guangxi University of Chinese Medicine to carry out research on the species and there was no endangered or protected species was involved in this study. The plant was identified as *U. macrophylla* by Professor Zhe-chen Qi of Zhejiang Sci-tech University. The total genomic DNA was extracted from its silica dried leaves using DNA Plantzol Reagent (Invitrogen, Carlsbad, CA) in accordance with the manufacturer’s instructions. The plastome sequences were generated using the Illumina HiSeq 2500 platform (Illumina Inc., San Diego, CA). In total, ca. 18.3 million high-quality clean reads (150 bp PE read length) were generated with adaptors trimmed. Aligning, assembly, and annotation were conducted by GetOrganelle v1.7.0c, BLAST, GeSeq (Michael et al. 2017) and GENEIOUS v11.0.5 (Biomatters Ltd., Auckland, New Zealand).

The full length of *U. macrophylla* cp sequence (GenBank accession no. OK534535) is 155,138 bp, consisting of a large single-copy (LSC with 85,750 bp) region, a small single-copy (SSC with 18,157 bp) region, and two inverted repeat regions (IR with 25,615 bp). The GC content of *U. macrophylla* cp genome was 37.5%. A total of 129 genes were contained in the genome (79 protein-coding genes, eight rRNA genes, and 37 tRNA genes). Seventeen genes had two copies, which were comprised of protein-coding genes (*ndhB*, *rpl2*, *rpl23*, *rps7*, and *ycf2*), seven tRNA genes (*trnA-UGC*, *trnI-CAU*, *trnI-GAU*, *trnL-CAA*, *trnN-GUU*, *trnR-ACG*, and *trnV-GAC*), and all four rRNA species (*rrn16*, *rrn23*, *rrn4.5*, and *rrn5*). In the genome, seven protein-coding genes (*ndhB*, *rpl2*, *rps16*, *atpF*, *rpoC1*, *rpl2*, and *ndhA*) had one intron, and two gene (*clpP*, *ycf3*) contained two introns.

To confirm the phylogenetic position of *Uncaria macrophylla*, we obtained 10 published cp genomes of Rubiaceae from NCBI, including *Galium aparine* (KY562587), *Paederia scandens* (MN567112), *Coffea arabica* (MK875244), *Coffea canephora* (NC030053), *Gardenia jasminoides* (NC057593), *Antirhea chinensis* (MK102723), *Cinchona officinalis* (MZ151891), *Mitragyna speciosa* (KY085908), *Neolamarckia cadamba* (MG572117), and *Uncaria rhynchophylla* (NC053701). *Asclepias nivea* from the family of Gentianales was used as outgroups. The sequence alignment was conducted using MAFFT v7.3 (Katoh and Standley 2013). The maximum-likelihood (ML) phylogenetic analyses were constructed using IQTREE v1.6.7 (Nguyen et al. 2015), with the best selected TVM + F+R3 model and 5000 bootstrap replicates. The phylogenetic tree revealed that *U. macrophylla* was closely related to *Uncaria rhynchophylla* (Miq.) Jacks (Rubinaceae) according to the current sampling extent (Figure 1).

**Figure 1.**
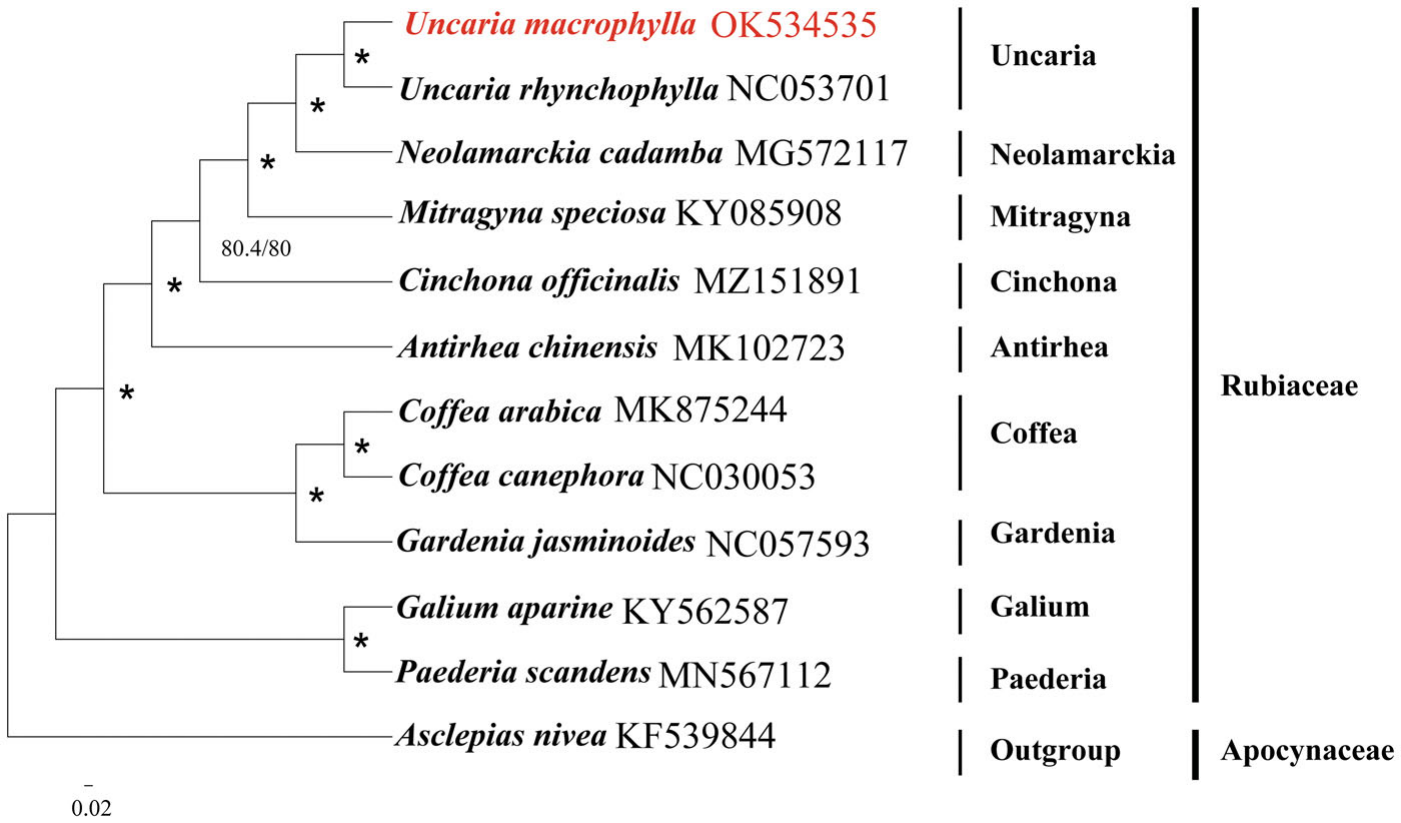
Phylogeny of *U. macrophylla* and other Rubiaceae based on complete chloroplast genomes. Accession numbers were listed behind each taxon. Statistical support values are shown on nodes. The ‘*’ approval rating for delegates is 100%.

## Data Availability

The genome sequence data that support the findings of this study are openly available in GenBank of NCBI (https://www.ncbi.nlm.nih.gov) under the accession no. OK534535. The associated BioProject, SRA, and Bio-Sample numbers are PRJNA769592, SRR16267456, and SAMN22147662, respectively.

## References

[CIT0001] Fujiwara H, Iwasaki K, Furukawa K, Seki T, He M, Maruyama M, Tomita N, Kudo Y, Higuchi M, Saido TC, et al. 2006. *Uncaria rhynchophylla*, a Chinese medicinal herb, has potent antiaggregation effects on Alzheimer's beta-amyloid proteins. J Neurosci Res. 84(2):427–433. 16676329 10.1002/jnr.20891

[CIT0002] Katoh K, Standley DM. 2013. MAFFT multiple sequence alignment software version 7: improvements in performance and usability. Mol Biol Evol. 30(4):772–780. 23329690 10.1093/molbev/mst010 PMC3603318

[CIT0004] Michael T, Pascal L, Tommaso P, Ulbricht-Jones ES, Axel F, Ralph B, Stephan G. 2017. GeSeq- versatile and accurate annotation of organelle genomes. Nucleic Acids Res. 45(W1):W6–W11. 28486635 10.1093/nar/gkx391 PMC5570176

[CIT0003] Nguyen N, Schmidt HA, Arndt VH, Quang MB. 2015. IQ-TREE: a fast and effective stochastic algorithm for estimating maximum-likelihood phylogenies. Mol Biol Evol. 32(1):268–274. 25371430 10.1093/molbev/msu300 PMC4271533

[CIT0005] Ndagijimana A, Wang X, Pan G, Zhang F, Feng H, Olaleye O. 2013. A review on indole alkaloids isolated from *Uncaria rhynchophylla* and their pharmacological studies. Fitoterapia. 86:35–47. 23376412 10.1016/j.fitote.2013.01.018

[CIT0006] Wang ZJ, Wu J, Guo W, Zhu YZ. 2019. TCTAP A-055 novel rhynchophylline analogue, Y396, improves endothelial malfunction induced by oxidative stress in diabetes. J Am Coll Cardiol. 73(15):S29.

